# Metaplastic Breast Carcinoma: Clinicopathological Parameters and Prognostic Profile

**DOI:** 10.7759/cureus.14347

**Published:** 2021-04-07

**Authors:** Saroona Haroon, Shamail Zia, Umme Aiman Shirazi, Omer Ahmed, Ishaq Azeem Asghar, Muhammad Asad Diwan, Anoshia Afzal, Muhammad Irfan, Syed Jawwad Ali, Atif A Hashmi

**Affiliations:** 1 Pathology, King's Mill Hospital - Sherwood Forest Hospitals, National Health Service (NHS) Foundation Trust, Ashfield, GBR; 2 Pathology, Prince Faisal Oncology Centre, King Fahad Specialist Hospital, Buraidah, SAU; 3 Pathology, Ziauddin University, Karachi, PAK; 4 Pathology, Liaquat National Hospital and Medical College, Karachi, PAK; 5 Internal Medicine, Liaquat National Hospital and Medical College, Karachi, PAK; 6 Pathology, Ascension St. John Hospital, Detroit, USA; 7 Pathology, Aga Khan University, Karachi, PAK; 8 Pathology, University of Oklahoma Health Sciences Center, Oklahoma City, USA; 9 Statistics, Liaquat National Hospital and Medical College, Karachi, PAK; 10 Pathology, Dow University of Health Sciences, Karachi, PAK

**Keywords:** metaplastic breast carcinoma, estrogen receptor, progesterone receptor, human epidermal growth factor receptor 2, immunohistochemistry, breast cancer

## Abstract

Introduction

Metaplastic breast carcinoma (MBC) is defined as breast cancer with a heterologous non-glandular component. MBC is considered a special type of breast cancer with a prognosis that is worse than invasive ductal carcinoma (IDC) of the breast. MBC is the most common breast cancer with a triple-negative profile. Therefore, in this study, we evaluated the clinicopathological parameters, recurrence and survival of MBC in our population.

Methods

We conducted a retrospective observational study in the Department of Histopathology at Prince Faisal Oncology Centre, Buraidah, Saudi Arabia, over a period of five years. All cases diagnosed as MBC were included in the study. Estrogen receptor (ER), progesterone receptor (PR), and human epidermal growth factor receptor 2 (HER2/neu) immunohistochemistry (IHC) was performed on representative tissue blocks.

Results

Total 183 cases of MBCs were included in the study, out of which 120 cases were excision specimens. The mean age of the patients was 48.84±12.99 years, and the most common age group was between 36 and 50 years of age. Most of the cases were tumor (T) stage T3 (50%), and nodal metastasis was present in 40% of cases. Most cases were grade III (78.7%). ER, PR and HER2/neu positivity was noted in 15.8%, 13.1%, and 9.8% cases, respectively. Follow-up data were available for 70 cases, with a median follow-up period of 4 (1-7) years. Tumor recurrence was noted in 31.4% cases, with a survival rate of 71.4%. Squamous, chondroid, spindle cell differentiation, and matrix production were noted in 70.5%, 7.1%, 13.7%, and 2.2% cases, respectively. A significant association of squamous differentiation was noted with HER2/neu positivity. An inverse association of spindle cell differentiation was seen with axillary metastasis. Survival analysis by Kaplan-Meier revealed a significant association of survival with tumor recurrence.

Conclusion

MBC is an important subtype of breast cancer, histopathological identification of which is challenging, owing to varied histological differentiation. We found squamous differentiation to be the most common in MBC, which was associated with HER2/neu positivity. A high recurrence rate of MBC was also observed in our study that was significantly associated with survival.

## Introduction

Metaplastic breast carcinoma (MBC) is defined as breast cancer with a heterologous non-glandular component [1]. The heterologous component can be in the form of squamous metaplasia or mesenchymal/sarcomatoid differentiation that resembles fibromatosis, fibrosarcoma, chondrosarcoma, osteosarcoma, rhabdomyosarcoma, spindle cells, matrix-producing, and angiosarcoma or in combination. MBC is considered a special type of breast cancer with a prognosis that is worse than invasive ductal carcinoma (IDC) of the breast. Moreover, MBC is mostly negative for estrogen receptor (ER), progesterone receptor (PR), and human epidermal growth factor receptor 2 (HER2/neu), and therefore, the treatment options are limited. In Pakistan, young-age breast cancers are common and the proportion of triple-negative breast cancer is alarmingly high [2,3]. MBC is the most common breast cancer with a triple-negative profile [4]. Therefore, in this study, we evaluated the clinicopathological parameters, recurrence, and survival of MBC in our population.

## Materials and methods

**Figure 1:**
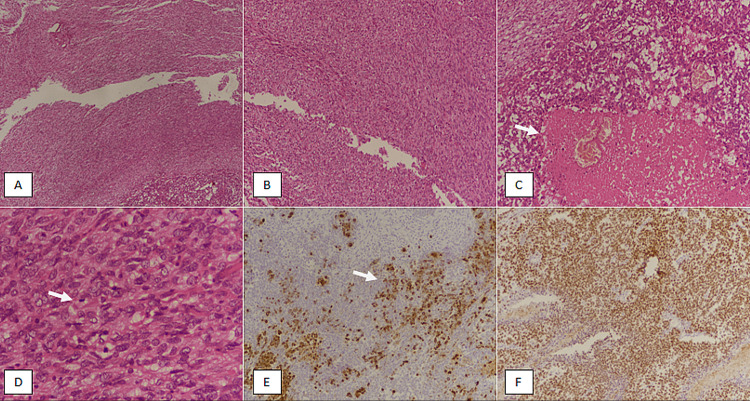
Metaplastic breast carcinoma with spindle cell (mesenchymal) differentiation. (A) H & E-stained section at 40x magnification showing sheets of spindle cell cells. (B) H & E-stained section at 100x magnification revealing atypical spindled tumor cells. (C) H & E-stained section of another area of tumor depicting epithelioid tumor cells with a central area of necrosis (arrow). (D) 400x magnification showing marked nuclear atypia with evident mitosis (arrow). (E) Pan-cytokeratin immunostain showing patchy positivity in the tumor (arrow). (F) p63 immunostaining revealing diffuse positivity in tumor cells. H & E, hematoxylin and eosin

**Figure 2:**
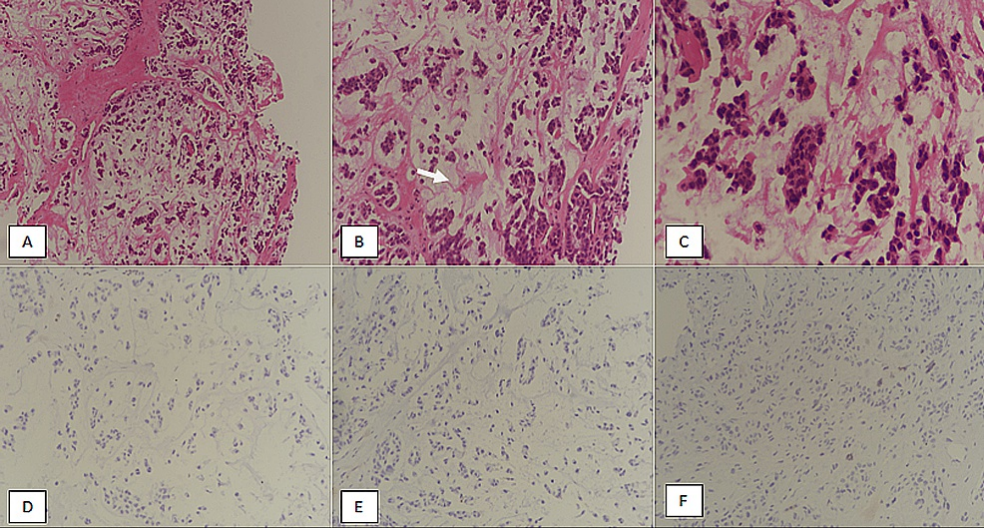
Metaplastic breast carcinoma, matrix-producing. (A) H & E-stained section at 100x magnification showing clusters of tumor cells with background matrix production. (B) H & E-stained section at 200x magnification revealing tumor cells and clusters with matrix production (arrow). (C) H & E-stained section at 400x magnification showing moderate to marked atypia. (D) ER immunostaining showing negativity in tumor cells. (E) PR immunostaining revealing absence of nuclear expression in tumor cells. (F) HER2/neu immunostaining showing absence of membranous positivity. H & E, hematoxylin and eosin; ER, estrogen receptor; PR, progesterone receptor; HER2/neu, human epidermal growth factor receptor 2

We conducted a retrospective observational study in the Department of Histopathology at Prince Faisal Oncology Centre, Buraidah, Saudi Arabia, over a period of five years. The specimens included were trucut biopsy, lumpectomy and mastectomy with or without axillary lymph node dissection. Cases with neoadjuvant chemotherapy before surgical excision were excluded from the study. The specimens were received in the histopathology lab and were grossed according to standard protocols [5,6]. Representative sections were taken from the tumor; resection margins and lymph nodes. Pathological parameters, such as tumor size and grade, were recorded. All cases diagnosed as MBC in the study period were included in the study. All slides and blocks were retrieved and histopathological diagnosis was reviewed by senior pathologists. The diagnosis of MBC was suspected on histology based on features including squamous differentiation, spindle cell (mesenchymal) differentiation, chondrosarcomatous/osteosarcomatous differentiation or matrix production. Pancytokeratin and p63 immunostains were performed to confirm the diagnosis of MBC (Figures 1A-1F, 2A-2F).

ER, PR and HER2/neu immunohistochemistry (IHC) was performed on representative tissue blocks. ER and PR nuclear expression of more than 1% was considered positive. Strong and complete membranous expression of HER2/neu in more than 10% tumor cells was taken as positive HER2/neu expression on IHC. For cases with equivocal HER2/neu IHC, fluorescence in situ hybridization (FISH) studies were performed to confirm the gene amplification, as performed in previous studies [7,8].

Data analysis was performed using Statistical Package for Social Sciences (Version 26.0, IBM Inc., Armonk, USA). Chi-square, independent t-test, and Fisher’s exact tests were used to check the association. Survival analysis was done by the Kaplan-Meier method. P-values < 0.05 were considered significant.

## Results

Total 183 cases of MBCs were included in the study, out of which 120 cases were excision specimens. The mean age of the patients was 48.84±12.99 years, and the most common age group was between 36 and 50 years of age. Most of the cases were tumor (T) stage T3 (50%), and nodal metastasis was present in 40% of cases. Most cases were grade III (78.7%). ER, PR, and HER2/neu positivity was noted in 15.8%, 13.1%, and 9.8% cases, respectively. Follow-up data were available for 70 cases, with a median follow-up period of 4 (1-7) years. Tumor recurrence was noted in 31.4% cases, with a survival rate of 71.4%. Squamous, chondroid, spindle cell differentiation, and matrix production were noted in 70.5%, 7.1%, 13.7%, and 2.2% cases, respectively (Table 1).

**Table 1:** Descriptive statistics of study population SD, standard deviation; T, tumor; N, nodal; ER, estrogen receptor; PR, progesterone receptor; HER2/neu, human epidermal growth factor receptor 2

Clinicopathological characteristics	Values
Age (years), mean±SD	48.84±12.99
Age groups	
≤35 years, n (%)	31 (16.9)
36-50 years, n (%)	81 (44.3)
>50 years, n (%)	71 (38.8)
Tumor size (cm), mean±SD	5.85±3.14
Follow-up (years), median (range)	4 (1–7)
Laterality	
Right breast, n (%)	88 (48.1)
Left breast, n (%)	95 (51.9)
Specimen type	
Trucut biopsy, n (%)	63 (34.4)
Modified radical mastectomy (MRM), n (%)	48 (26.2)
Breast conservation surgery, n (%)	37 (20.2)
Simple mastectomy, n (%)	35 (19.1)
T-stage (n=120)	
T1, n (%)	10 (8.3)
T2, n (%)	50 (41.7)
T3, n (%)	60 (50)
Axillary metastasis (n=120)	
Present, n (%)	48 (40)
Absent, n (%)	72 (60)
N-stage (n=120)	
N0, n (%)	72 (60)
N1, n (%)	26 (21.7)
N2, n (%)	13 (10.8)
N3, n (%)	9 (7.5)
Grade	
Grade I, n (%)	1 (0.5)
Grade II, n (%)	38 (20.8)
Grade III, n (%)	144 (78.7)
ER	
Positive, n (%)	29 (15.8)
Negative, n (%)	154 (84.2)
PR	
Positive, n (%)	24 (13.1)
Negative, n (%)	159 (86.9)
HER2/neu	
Positive, n (%)	18 (9.8)
Negative, n (%)	165 (90.2)
Recurrence (n=70)	
Yes, n (%)	22 (31.4)
No, n (%)	48 (68.6)
Survival status (n=70)	
Alive, n (%)	50 (71.4)
Expired, n (%)	20 (28.6)
Squamous differentiation	
Present, n (%)	129 (70.5)
Absent, n (%)	54 (29.5)
Chondroid differentiation	
Present, n (%)	13 (7.1)
Absent, n (%)	170 (92.9)
Spindle cell differentiation	
Present, n (%)	25 (13.7)
Absent, n (%)	158 (86.3)
Matrix production	
Present, n (%)	4 (2.2)
Absent, n (%)	179 (97.8)

Table 2 shows the association of squamous differentiation with clinicopathological and prognostic parameters. A significant association of squamous differentiation was noted with HER2/neu positivity. Cases with squamous differentiation revealed a higher frequency of HER2/neu positivity.

**Table 2:** Association of squamous differentiation with clinicopathological features *Independent t-test was applied, **Chi-square test was applied, ***Fisher’s exact test was applied, ****p-value significant as <0.05 SD, standard deviation; T, tumor; N, nodal; ER, estrogen receptor; PR, progesterone receptor; HER2/neu, human epidermal growth factor receptor 2

Clinicopathological features	Values		P-value
	Squamous differentiation		
	Present	Absent	
Age (years)*, mean±SD	48.84±10.83	48.84±13.32	0.999
Age groups**			
≤35 years, n (%)	22 (17.1)	9 (16.7)	0.559
36-50 years, n (%)	54 (41.9)	27 (50)	
>50 years, n (%)	53 (41.1)	18 (33.3)	
Tumor size (cm)*, mean±SD	5.93±2.67	5.83±3.21	0.913
T-stage (n=120)**			
T1, n (%)	9 (11.7)	1 (2.3)	0.163
T2, n (%)	29 (37.7)	21 (48.8)	
T3, n (%)	39 (50.6)	21 (48.8)	
Axillary metastasis (n=120)**			
Present, n (%)	31 (40.3)	17 (39.5)	0.938
Absent, n (%)	46 (59.7)	26 (60.5)	
N-stage (n=120)***			
N0, n (%)	46 (59.7)	26 (60.5)	0.314
N1, n (%)	14 (18.2)	12 (27.9)	
N2, n (%)	9 (11.7)	4 (9.3)	
N3, n (%)	8 (10.4)	1 (2.3)	
Grade**			
Grade I, n (%)	1 (0.8)	0 (0)	0.520
Grade II, n (%)	24 (18.6)	14 (25.9)	
Grade III, n (%)	104 (80.6)	40 (74.1)	
ER**			
Positive, n (%)	20 (15.5)	9 (16.7)	0.844
Negative, n (%)	109 (84.5)	45 (83.3)	
PR**			
Positive, n (%)	19 (14.7)	5 (9.3)	0.317
Negative, n (%)	110 (85.3)	49 (90.7)	
HER2/neu**			
Positive, n (%)	18 (14)	0 (0)	0.004****
Negative, n (%)	111 (86)	54 (100)	
Recurrence (n=70)***			
Yes, n (%)	18 (37.5)	4 (18.2)	0.165
No, n (%)	30 (62.5)	18 (81.8)	
Survival status (n=70)***			
Alive, n (%)	32 (66.7)	18 (81.8)	0.259
Expired, n (%)	16 (33.3)	4 (18.2)	

Table 3 depicts the association of chondroid differentiation with pathological and prognostic factors; however, no significant association of chondroid differentiation was noted with clinicopathological parameters.

**Table 3:** Association of chondroid differentiation with clinicopathological features *Independent t-test was applied, **Fisher’s exact test was applied SD, standard deviation; T, tumor; N, nodal; ER, estrogen receptor; PR, progesterone receptor; HER2/neu, human epidermal growth factor receptor

Clinicopathological features	Values		P-value
	Chondroid differentiation		
	Yes	No	
Age (years)*, mean±SD	46.53±11.97	49.01±13.08	0.509
Age groups**			
≤35 years, n (%)	3 (23.1)	28 (16.5)	0.435
36-50 years, n (%)	7 (53.8)	74 (43.5)	
>50 years, n (%)	3 (23.1)	68 (40)	
Tumor size (cm)*, mean±SD	6.51±3.58	5.79±3.10	0.490
T-stage (n=120)**			
T1, n (%)	0 (0)	10 (9.1)	0.519
T2, n (%)	6 (60)	44 (40)	
T3, n (%)	4 (40)	56 (50.9)	
Axillary metastasis (n=120)**			
Present, n (%)	3 (30)	45 (40.9)	0.738
Absent, n (%)	7 (70)	65 (59.1)	
N-stage (n=120)**			
N0, n (%)	7 (7)	65 (59.1)	1.000
N1, n (%)	2 (20)	24 (21.8)	
N2, n (%)	1 (10)	12 (10.9)	
N3, n (%)	0 (0)	9 (8.2)	
Grade**			
Grade I, n (%)	0 (0)	1 (0.6)	0.100
Grade II, n (%)	6 (46.2)	32 (18.8)	
Grade III, n (%)	7 (53.8)	137 (80.6)	
ER**			
Positive, n (%)	2 (15.4)	27 (15.9)	1.000
Negative, n (%)	11 (84.6)	143 (84.1)	
PR**			
Positive, n (%)	2 (15.4)	22 (12.9)	0.681
Negative, n (%)	11 (84.6)	148 (87.1)	
HER2/neu**			
Positive, n (%)	0 (0)	18 (10.6)	0.370
Negative, n (%)	13 (100)	152 (89.4)	
Recurrence (n=70)**			
Yes, n (%)	2 (50)	20 (30.3)	0.585
No, n (%)	2 (50)	46 (69.7)	
Survival status (n=70)**			
Alive, n (%)	4 (100)	46 (69.7)	0.318
Expired, n (%)	0 (0)	20 (30.3)	

An inverse association of spindle cell differentiation was seen with axillary metastasis. Cases of MBC with spindle cell differentiation revealed the lower frequency of axillary metastasis than MBC without spindle cell differentiation (Table 4).

**Table 4:** Association of spindle cell differentiation with clinicopathological features *Independent t-test was applied, **Chi-square test was applied, ***Fisher’s exact test was applied, ****p-value significant as <0.05 SD, standard deviation; T, tumor; N, nodal; ER, estrogen receptor; PR, progesterone receptor; HER2/neu, human epidermal growth factor receptor

Clinicopathological features	Values		P-value
	Spindle cell differentiation		
	Yes	No	
Age (years)*, mean±SD	48.84±10.83	48.84±13.32	0.999
Age groups***			
≤35 years, n (%)	2 (8)	29 (18.4)	0.344
36-50 years, n (%)	14 (56)	67 (42.4)	
>50 years, n (%)	9 (36)	62 (39.2)	
Tumor size (cm)*, mean±SD	5.93±2.67	5.83±3.21	0.913
T-stage (n=120)***			
T1, n (%)	1 (6.7)	9 (8.6)	0.833
T2, n (%)	5 (33.3)	45 (42.9)	
T3, n (%)	9 (60)	51 (48.6)	
Axillary metastasis (n=120)**			
Present, n (%)	2 (13.3)	46 (43.8)	0.024****
Absent, n (%)	13 (86.7)	59 (56.2)	
N-stage (n=120)***			
N0, n (%)	13 (86.7)	59 (56.2)	0.174
N1, n (%)	2 (13.3)	24 (22.9)	
N2, n (%)	0 (0)	13 (12.4)	
N3, n (%)	0 (0)	9 (8.6)	
Grade***			
Grade I, n (%)	0 (0)	1 (0.6)	0.161
Grade II, n (%)	9 (36)	29 (18.4)	
Grade III, n (%)	16 (64)	128 (81)	
ER***			
Positive, n (%)	2 (8)	27 (17.1)	0.378
Negative, n (%)	23 (92)	131 (82.9)	
PR***			
Positive, n (%)	0 (0)	24 (15.2)	0.049****
Negative, n (%)	25 (100)	134 (84.8)	
HER2/neu***			
Positive, n (%)	0 (0)	18 (11.4)	0.139
Negative, n (%)	25 (100)	140 (88.6)	
Recurrence (n=70)***			
Yes, n (%)	2 (25)	20 (32.3)	1.000
No, n (%)	6 (75)	42 (67.7)	
Survival status (n=70)***			
Alive, n (%)	4 (50)	46 (74.2)	0.212
Expired, n (%)	4 (50)	16 (25.8)	

Table 5 shows the association of matrix-producing MBC with prognostic and pathological parameters, however, no significant association of matrix production was noted with clinicopathological features.

**Table 5:** Association of matrix production with clinicopathological features *Independent t-test was applied, **Fisher’s exact test was applied SD, standard deviation; T, tumor; N, nodal; ER, estrogen receptor; PR, progesterone receptor; HER2/neu, human epidermal growth factor receptor

Clinicopathological features	Values		P-value
	Matrix production		
	Yes	No	
Age (years)*, mean±SD	43.75±5.31	48.95±13.09	0.430
Age groups**			
≤35 years, n (%)	0 (0)	31 (17.3)	0.133
36-50 years, n (%)	4 (100)	77 (43)	
>50 years, n (%)	0 (0)	71 (39.7)	
Tumor size (cm)*, mean±SD	6.65±4.91	5.82±3.09	0.606
T-stage (n=120)**			
T1, n (%)	0 (0)	10 (8.6)	0.528
T2, n (%)	3 (75)	47 (40.5)	
T3, n (%)	1 (25)	59 (50.9)	
Axillary metastasis (n=120)**			
Present, n (%)	2 (50)	46 (39.7)	1.000
Absent, n (%)	2 (50)	70 (60.3)	
N-stage (n=120)**			
N0, n (%)	2 (50)	70 (60.3)	0.581
N1, n (%)	2 (50)	24 (20.7)	
N2, n (%)	0 (0)	13 (11.2)	
N3, n (%)	0 (0)	9 (7.8)	
Grade**			
Grade I, n (%)	0 (0)	1 (0.6)	1.000
Grade II, n (%)	1 (25)	37 (20.7)	
Grade III, n (%)	3 (75)	141 (78.8)	
ER**			
Positive, n (%)	2 (50)	27 (15.1)	0.119
Negative, n (%)	2 (50)	152 (84.9)	
PR**			
Positive, n (%)	2 (50)	22 (12.3)	0.084
Negative, n (%)	2 (50)	157 (87.7)	
HER2/neu**			
Positive, n (%)	0 (0)	18 (10.1)	1.000
Negative, n (%)	4 (100)	161 (89.9)	
Recurrence (n=70)**			
Yes, n (%)	0 (0)	22 (32.4)	1.000
No, n (%)	2 (100)	46 (67.6)	
Survival status (n=70)**			
Alive, n (%)	2 (100)	48 (70.6)	1.000
Expired, n (%)	0 (0)	20 (29.4)	

**Figure 3:**
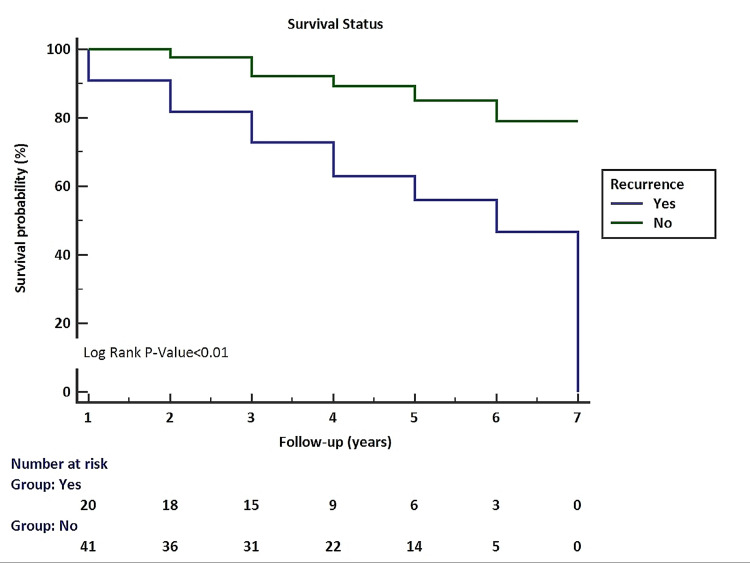
Association of tumor recurrence with survival

Survival analysis by Kaplan-Meier revealed a significant association of survival with tumor recurrence. Patients of MBC with recurrence had lower survival than cases without tumor recurrence (Figure 3).

## Discussion

MBC is an aggressive breast cancer type that accounts for approximately one percent of invasive breast cancer [9]. The neoplastic epithelium tends to differentiate into various heterologous non-glandular components such as metaplastic squamous cells and mesenchymal elements with sarcomatoid, rhabdoid, spindle, or other morphological patterns [9]. MBCs are usually larger in size at the time of diagnosis compared with IDC and other breast cancer subtypes and typically have a poor prognosis [9,10]. MBCs lack hormone receptors (ER, PR) and HER2/neu expression and are therefore difficult to treat [3,4,9,10]. MBCs have almost two times higher rates of recurrence than other triple-negative breast cancers and a shorter overall disease-free survival [11]. Previous literature has mentioned that MBC responds poorly to various chemotherapeutic regimens [10,11].

Nowara et al. found that adjuvant radiation therapy (in addition to standard chemotherapy) was associated with significantly improved survival and recommended further studies on a larger group [12]. Beatty et al. found that even though MBCs had overall worse prognostic features, aggressive multidisciplinary management was associated with comparable outcomes between MBC and conventional breast cancer cases [13]. Pezzi et al. reported that MBCs tend to involve axillary lymph nodes less commonly but metastasize to distant locations more frequently [14]. Chen et al. reported poor response to several chemotherapeutic agents with a relatively better response to taxane-based chemotherapy [15]. McCart Reed et al. reported that loss of cytokeratin expression, overexpression of epidermal growth factor receptor (EGFR) and the coexistence of multiple morphological patterns were associated with poor prognosis in addition to the previously described prognostic factors such as tumor size and grade [16]. Cimino-Mathews et al. compared multiple studies and further reported an overall poor prognosis of MBC despite aggressive therapy and stated that MBCs express high levels of vascular/angiogenesis markers like vascular endothelial growth factor and suggested targeted therapy trials in future studies [17].

MBCs are a heterogeneous group of tumors including both low-grade and prognostically better tumors, along with high-grade and prognostically poor tumors. The biological behavior of these tumors correlates with tumor grade. The low-grade adenosquamous and fibromatosis-like carcinomas are associated with indolent clinical behavior [18]. Alternatively, high-grade adenosquamous carcinoma and high-grade spindle cell carcinomas are associated with a poor prognosis [19]. Matrix-producing MBCs are also associated with a relatively better prognosis than other high-grade subtypes of MBC [19]. We also noted that the matrix production was inversely associated with axillary metastasis.

In our study, mean tumor size was 5.85±3.14 cm. Previous studies have emphasized that MBC tends to have larger tumor size (higher T-stage), and a lower incidence of axillary metastasis (lower N-stage) than IDC [20]. As MBCs tend to present at a higher T-stage, a significant subset of patients require neoadjuvant chemotherapy. However, MBCs are associated with a poor response to neoadjuvant chemotherapy with a complete pathological response rate of 10%-17% [21].

The main differential diagnosis of spindle cell MBC is malignant phyllodes tumor (MPT). However, MPT typically has a leaf-like architecture with benign slit-like entrapped epithelial elements. In difficult cases, immunohistochemistry with pancytokeratin, p63, and CD34 is helpful. While MBCs are positive (at least focally) with pancytokeratin and p63, MPTs show positive expression with CD34.

Our study had few limitations, including small sample size and single-institution data, but showed overall comparable results with significant association of squamous differentiation with HER2/neu expression and recurrence with survival. More clinical trials are required with targeted therapies to determine treatment response and survival improvement.

## Conclusions

MBC is an aggressive subtype of breast cancer and its histopathological identification is essential as it displays unique clinicopathological characteristics, including the lack of expression of hormone receptors and HER2/neu. We noted that squamous differentiation is the most common histological pattern, and squamous differentiation was significantly associated with HER2/neu positivity in MBC in our study. Moreover, spindle cell differentiation was inversely associated with axillary metastasis. A high frequency of tumor recurrence was noted in our study in patients with MBC, and recurrence was also noted to be significantly associated with the patient’s survival.

## References

[1] Hashmi AA, Aijaz S, Mahboob R (2018). Clinicopathologic features of invasive metaplastic and micropapillary breast carcinoma: comparison with invasive ductal carcinoma of breast. BMC Res Notes.

[2] Hashmi AA, Naz S, Hashmi SK (2019). Epidermal growth factor receptor (EGFR) overexpression in triple-negative breast cancer: association with clinicopathologic features and prognostic parameters. Surg and Exp Pathol.

[3] Hashmi AA, Naz S, Hashmi SK (2018). Prognostic significance of p16 & p53 immunohistochemical expression in triple negative breast cancer. BMC Clin Pathol.

[4] Hashmi AA, Edhi MM, Naqvi H, Faridi N, Khurshid A, Khan M (2014). Clinicopathologic features of triple negative breast cancers: an experience from Pakistan. Diagn Pathol.

[5] Hashmi AA, Iftikhar SN, Haider R, Haider R, Irfan M, Ali J (2020). Solid papillary carcinoma of breast: clinicopathologic comparison with conventional ductal carcinoma of breast. Cureus.

[6] Hashmi AA, Iftikhar SN, Munawar S, Shah A, Irfan M, Ali J (2020). Encapsulated papillary carcinoma of breast: clinicopathological features and prognostic parameters. Cureus.

[7] Hashmi AA, Munawar S, Rehman N (2021). Invasive papillary carcinoma of the breast: clinicopathological features and hormone receptor profile. Cureus.

[8] Hashmi A A, Zia S, Yaqeen S (2021). Mucinous breast carcinoma: clinicopathological comparison with invasive ductal carcinoma. Cureus.

[9] Budzik MP, Patera J, Sobol M, Czerw AI, Deptała A, Badowska-Kozakiewicz AM (2019). Clinicopathological characteristics of metaplastic breast cancer - analysis of the basic immunohistochemical profile and comparison with other invasive breast cancer types. Breast.

[10] Lee H, Jung SY, Ro JY (2012). Metaplastic breast cancer: clinicopathological features and its prognosis. J Clin Pathol.

[11] Reddy TP, Rosato RR, Li X, Moulder S, Piwnica-Worms H, Chang JC (2020). A comprehensive overview of metaplastic breast cancer: clinical features and molecular aberrations. Breast Cancer Res.

[12] Nowara E, Drosik A, Samborska-Plewicka M, Nowara EM, Stanek-Widera A (2014). Metaplastic breast carcinomas - analysis of prognostic factors in a case series. Contemp Oncol (Pozn).

[13] Beatty JD, Atwood M, Tickman R, Reiner M (2006). Metaplastic breast cancer: clinical significance. Am J Surg.

[14] Pezzi CM, Patel-Parekh L, Cole K, Franko J, Klimberg VS, Bland K (2007). Characteristics and treatment of metaplastic breast cancer: analysis of 892 cases from the National Cancer Data Base. Ann Surg Oncol.

[15] Chen IC, Lin CH, Huang CS (2011). Lack of efficacy to systemic chemotherapy for treatment of metaplastic carcinoma of the breast in the modern era. Breast Cancer Res Treat.

[16] McCart Reed AE, Kalaw E, Nones K (2019). Phenotypic and molecular dissection of metaplastic breast cancer and the prognostic implications. J Pathol.

[17] Cimino-Mathews A, Verma S, Figueroa-Magalhaes MC (2016). A clinicopathologic analysis of 45 patients with metaplastic breast carcinoma. Am J Clin Pathol.

[18] Rakha EA, Badve S, Eusebi V (2016). Breast lesions of uncertain malignant nature and limited metastatic potential: proposals to improve their recognition and clinical management. Histopathology.

[19] Rakha EA, Tan PH, Varga Z (2015). Prognostic factors in metaplastic carcinoma of the breast: a multi-institutional study. Br J Cancer.

[20] Ong CT, Campbell BM, Thomas SM (2018). Metaplastic breast cancer treatment and outcomes in 2500 patients: a retrospective analysis of a National Oncology Database. Ann Surg Oncol.

[21] Al-Hilli Z, Choong G, Keeney MG, Visscher DW, Ingle JN, Goetz MP, Jakub JW (2019). Metaplastic breast cancer has a poor response to neoadjuvant systemic therapy. Breast Cancer Res Treat.

